# Surgical Excision of Lipomas in Bilateral Upper Limbs and Right Thigh: A Case Report

**DOI:** 10.7759/cureus.53317

**Published:** 2024-01-31

**Authors:** Akanksha Yachmaneni, Suhas Jajoo, Chandrashekhar Mahakalkar, Shivani Kshirsagar, Shubham Durge

**Affiliations:** 1 General Surgery, Jawaharlal Nehru Medical College, Datta Meghe Institute of Higher Education & Research, Wardha, IND

**Keywords:** individualized treatment, benign neoplasms, symptomatic lipomas, soft tissue tumours, surgical excision, lipomas

## Abstract

Lipomas are common benign soft tissue tumors composed primarily of mature adipose tissue. They are often encountered clinically due to their characteristic slow growth, typically as soft, painless, subcutaneous nodules. While lipomas are generally asymptomatic, surgical intervention is sought by patients when they lead to discomfort, cosmetic concerns, or functional impairment. In this case report, we present the successful surgical excision of 25 lipomas in the bilateral upper limbs and right thigh of a 43-year-old male. Pre-operative assessment, precise surgical technique, and post-operative care are highlighted as essential management components. The case emphasizes the importance of individualized treatment, ensuring symptomatic lipomas' diagnosis and effective management. This report serves as a valuable reference for healthcare professionals caring for patients with lipomatous lesions, contributing to understanding soft tissue tumor management.

## Introduction

Lipomas, benign soft tissue tumors composed primarily of mature adipose tissue, represent one of the most prevalent neoplasms within the spectrum of soft tissue tumors [1]. These slow-growing, non-invasive lesions typically manifest as soft, mobile, and painless subcutaneous nodules. Lipomas can occur at various anatomical sites throughout the body, with the most common locations being the trunk, upper limbs, and neck [2]. The exact etiology of lipomas remains elusive, but factors such as genetic predisposition and metabolic disturbances have been implicated in their development [3]. While lipomas are generally asymptomatic and may not require immediate intervention, patients often seek surgical treatment due to cosmetic concerns or discomfort caused by the enlargement and compression of adjacent structures [4]. In rare cases, lipomas can attain considerable sizes, leading to functional impairment or pain. Their slow but relentless growth can lead to aesthetic and physical concerns for patients.

Surgical excision remains the mainstay of treatment, offering an effective solution for symptomatic lipomas [5]. This procedure can remove bothersome lipomas and provide a histopathological confirmation of the diagnosis, ensuring the absence of malignancy or other underlying conditions [6]. The success of surgical excision is highly dependent on precise pre-operative planning, including selecting the most appropriate surgical technique and anesthetic modality, along with meticulous intra-operative execution [7]. This case report describes the surgical management of multiple lipomas in the bilateral upper limbs and right thigh of a 43-year-old male, highlighting the significance of accurate diagnosis, patient selection, and surgical technique for a favorable outcome. This report aims to shed light on the comprehensive approach to the surgical management of lipomas and the importance of tailoring the treatment to each patient's needs.

## Case presentation

A 43-year-old male presented to the outpatient department of a tertiary care hospital with complaints of swelling in his hands and right thigh. During the patient history collection, he disclosed that the swelling had progressively increased over the past 12 years. He had been referred to the tertiary care hospital by a physician he met during a medical camp in his village.

**Figure 1:**
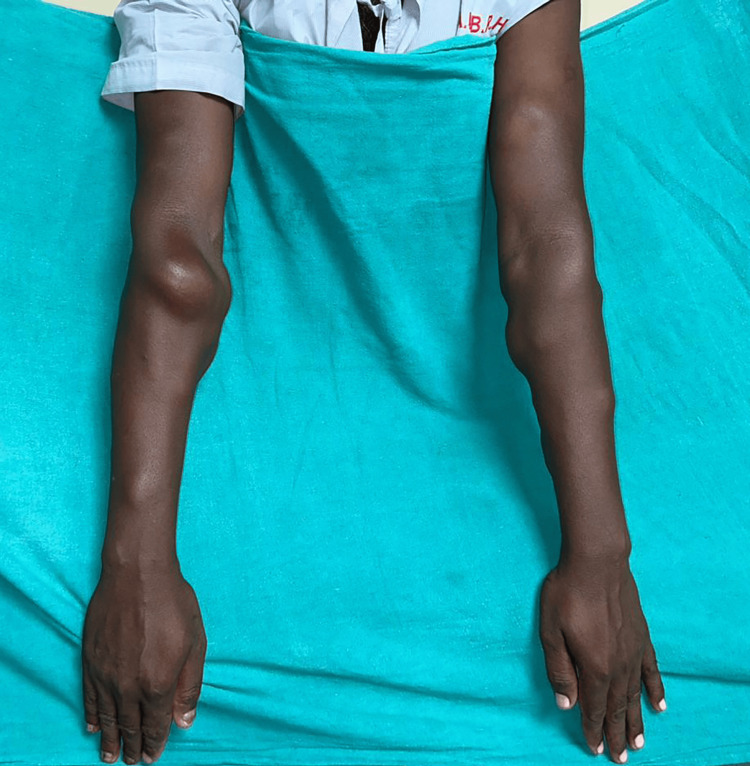
Image showing the presence of lipomas on the upper limbs

**Figure 2:**
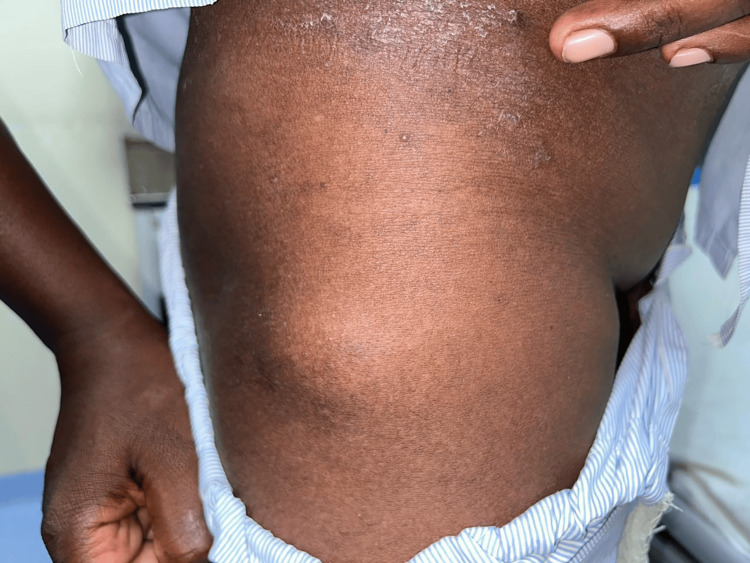
Image showing the presence of multiple lipomas on the lateral side of the right thigh

Upon physical examination, the healthcare provider noted the presence of multiple lipomas on both upper limbs and the lateral side of the right thigh (Figures 1, 2). The giant lipoma on one of his upper limbs measured 8 x 3 centimeters, and there was no evidence of pus discharge upon palpation. Consequently, the patient was advised to undergo blood tests, X-ray, and ultrasonography (USG).

USG revealed multiple lipomas on both upper limbs, with the largest one measuring 8 x 3 centimeters, and lipomas on the lateral side of the right thigh. Based on these findings, the decision was made to admit the patient to the surgical ward for further management. Pre-operative assessments were conducted, and the patient was deemed suitable for surgery. Under general anesthesia, the surgical team performed excision of multiple lipomas from both the upper limbs and the right thigh. The procedure involved making an S-shaped incision along the swelling, dissecting the margins of the lipoma to access it, and removing the lipoma in its entirety. Subcutaneous closure was achieved using Vicryl 2-0 for the deeper tissues and Ethilon 2-0 for the skin. Hemostasis was successfully maintained throughout the surgery.

**Figure 3:**
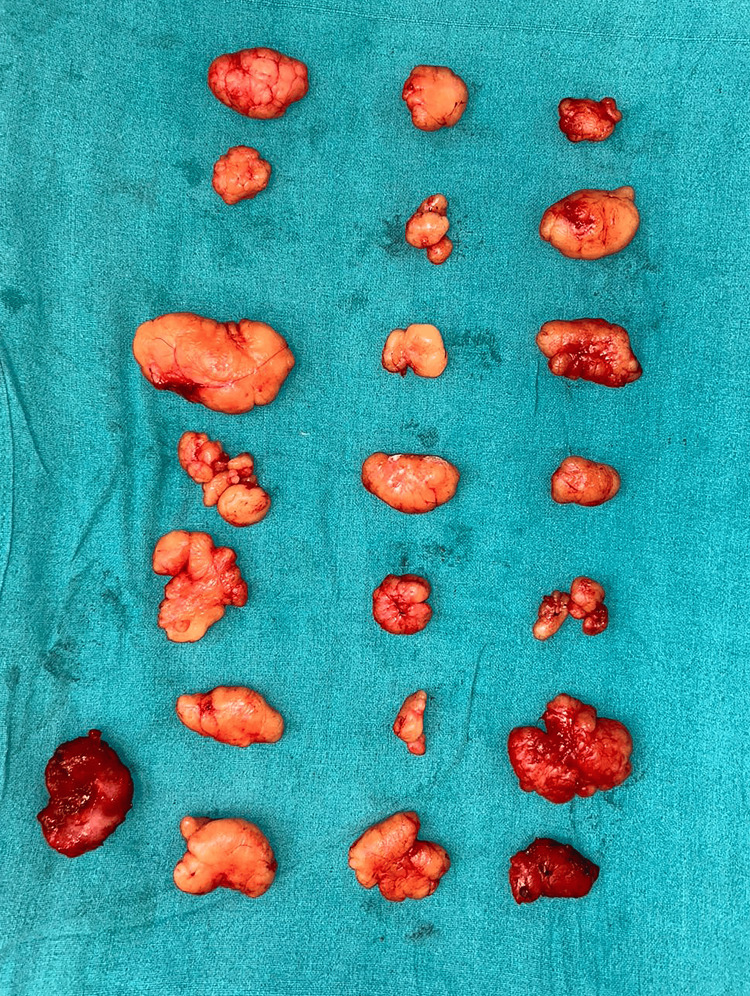
Image showing the lipomas after the removal from the patient

**Figure 4:**
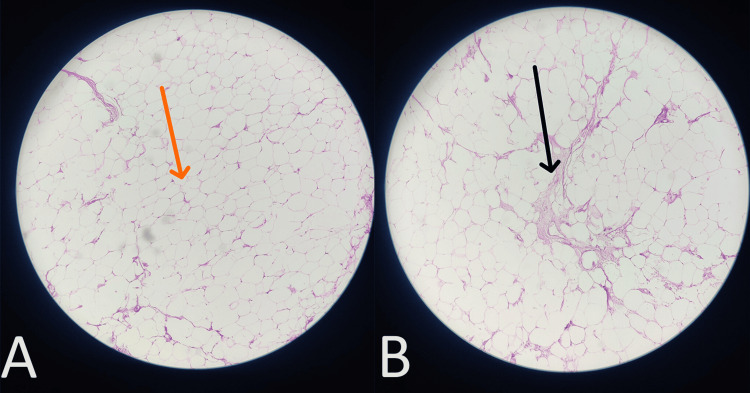
Image (magnification 40X) showing (A) proliferation of mature adipocytes and (B) skeletal muscle fibers infiltrated between mature adipocytes

Seven lipomas were removed from the left upper limb, 16 from the right upper limb, and two from the right thigh (Figure 3). Two lipoma specimens were sent for histopathological analysis, revealing that the proliferation of mature adipocytes (Figure 4A) and skeletal muscle fibers are infiltrated between mature adipocytes (Figure 4B). Following the surgery, the patient was transferred to the post-operative intensive care unit (ICU) for monitoring and recovery.

The post-operative dressing was regularly checked and changed, with no evidence of leakage or issues with the suture line; the wound was healing well. The patient's vital signs and hemodynamic stability remained satisfactory, leading to his eventual discharge.

## Discussion

Lipomas are benign soft tissue tumors characterized by slow growth and often encountered in clinical practice. Their typical clinical presentation as soft, mobile, painless nodules often leads to the decision of surgical removal in cases where they cause discomfort, cosmetic concerns, or functional impairment [8]. Surgical excision is considered the most effective and definitive treatment option for lipomas, as it addresses the patient's concerns and provides an opportunity for histopathological examination to confirm the diagnosis and exclude other underlying pathologies [3]. In this case, the choice of surgical technique, general anesthesia, and meticulous intra-operative procedures led to the successful removal of 25 lipomas spread across the bilateral upper limbs and right thigh without any complications. The surgical approach was tailored to the patient's specific distribution of lipomas.

One of the critical aspects of this case is the importance of pre-operative assessment and accurate diagnosis. USG played a vital role in confirming the presence of lipomas and evaluating their size and distribution. This information was critical in planning the surgical procedure and ensuring adequate patient counselling [9]. The decision to send two lipoma specimens for histopathological analysis post-surgery is best practice to confirm the diagnosis and rule out malignancy. Furthermore, post-operative care and monitoring are essential components of the surgical management of lipomas. In this case, post-operative dressing changes and monitoring were carried out diligently, leading to a complication-free recovery and the eventual discharge of the patient. This demonstrates the importance of meticulous wound care and post-operative follow-up to ensure optimal outcomes. As the case report illustrates, timely surgical intervention can provide functional and aesthetic benefits for patients with lipomas. While lipomas are generally considered benign, the potential for enlargement and compression of adjacent structures, as observed in this case, can lead to considerable discomfort and functional impairment. Therefore, surgical excision, as performed in this case, remains a safe and effective treatment option, with a high likelihood of a successful outcome [10].

## Conclusions

In conclusion, the surgical excision of multiple lipomas in the bilateral upper limbs and right thigh described in this case report highlights the importance of accurate diagnosis, meticulous surgical technique, and comprehensive post-operative care. It is a valuable reference for healthcare professionals and surgeons managing patients with symptomatic lipomas and emphasizes the significance of individualized treatment plans tailored to the patient's unique presentation.
